# Emotional brain network decoded by biological spiking neural network

**DOI:** 10.3389/fnins.2023.1200701

**Published:** 2023-07-11

**Authors:** Hubo Xu, Kexin Cao, Hongguang Chen, Awuti Abudusalamu, Wei Wu, Yanxue Xue

**Affiliations:** ^1^National Institute on Drug Dependence and Beijing Key Laboratory of Drug Dependence, Peking University, Beijing, China; ^2^Department of Pharmacology, School of Basic Medical Sciences, Peking University, Beijing, China; ^3^NHC Key Laboratory of Mental Health (Peking University), National Clinical Research Center for Mental Disorders (Peking University Sixth Hospital), Peking University Institute of Mental Health, Peking University Sixth Hospital, Beijing, China; ^4^State Key Laboratory of Multimodal Artificial Intelligence Systems, Institute of Automation, Chinese Academy of Sciences, Beijing, China; ^5^Chinese Institute for Brain Research, Beijing, China; ^6^Key Laboratory for Neuroscience, Ministry of Education/National Health Commission, Peking University, Beijing, China

**Keywords:** emotion, affective computing, brain network, neural oscillation, neuroregulation, self-backpropagation, spiking neural network, brain-computer interface

## Abstract

**Introduction:**

Emotional disorders are essential manifestations of many neurological and psychiatric diseases. Nowadays, researchers try to explore bi-directional brain-computer interface techniques to help the patients. However, the related functional brain areas and biological markers are still unclear, and the dynamic connection mechanism is also unknown.

**Methods:**

To find effective regions related to different emotion recognition and intervention, our research focuses on finding emotional EEG brain networks using spiking neural network algorithm with binary coding. We collected EEG data while human participants watched emotional videos (fear, sadness, happiness, and neutrality), and analyzed the dynamic connections between the electrodes and the biological rhythms of different emotions.

**Results:**

The analysis has shown that the local high-activation brain network of fear and sadness is mainly in the parietal lobe area. The local high-level brain network of happiness is in the prefrontal-temporal lobe-central area. Furthermore, the α frequency band could effectively represent negative emotions, while the α frequency band could be used as a biological marker of happiness. The decoding accuracy of the three emotions reached 86.36%, 95.18%, and 89.09%, respectively, fully reflecting the excellent emotional decoding performance of the spiking neural network with self- backpropagation.

**Discussion:**

The introduction of the self-backpropagation mechanism effectively improves the performance of the spiking neural network model. Different emotions exhibit distinct EEG networks and neuro-oscillatory-based biological markers. These emotional brain networks and biological markers may provide important hints for brain-computer interface technique exploration to help related brain disease recovery.

## Introduction

Emotion influences human rational decision-making, behavior, and cognition and is indispensable for social communication. Studying neural circuit mechanisms of emotion is integral to “Brain Science and Brain-Like Intelligence Technology of China” (Poo et al., 2016). Many mental disorders and diseases are related to abnormal emotional regulation. With a rapid development of computer science, psychology, and neuroscience, affective computing attracts more and more research interest. Efficiency decoding of EEG signals is significant in studying affective computing (Seeber et al., 2019). Neural networks (NN) are widely used in affective computing because of their high decoding accuracy (Kohn et al., 2014). In recent years, NN has continued to be improved in structure, depth, and complexity, and the decoding accuracy of EEG signals reaches a high level with the advantage of high-performance graphics computers (Mousa and Hussein, 2021). The process of affection computing is to extract EEG features first, then feed them into an NN for classification. The advantage of this method is that it takes emotion-related features as input, reduces the dimension of input data, and reduces the difficulty and instability of training the prediction model. Through the analysis of the learning content of the NN, such as the classification weight of different features, the critical features in emotion recognition can be explained, such as frequency band, channel, and brain area. The disadvantage of this method is that it extracts features first, which reduces the information entropy of the original EEG signal related to emotion, and this process is irreversible. Another method is to take original EEG data as the neural network input. This method makes full use of the autonomous learning ability of the neural network to learn the information and patterns related to emotion from the original EEG. However, this method also has its disadvantages. The information and patterns learned by NN are challenging to explain, which limits the exploration and research of emotional neural activities (Liu et al., 2021). Given these problems, it is significant to design an NN based on the characteristics of emotion-related EEG signals to learn the neural activity information.

The spiking neural network (SNN) is the third-generation neural network, and the neuron emits a signal by spike. Compared with the traditional NN, the information transmission of the SNN is based on the binary signal to encode the information. It is also more conducive to simulating the neurons' plasticity (Ghosh-Dastidar and Adeli, 2009). However, the training progress of the SNN model was mainly forward propagation and could not iteratively optimize the calculation error. The hippocampal neuron was found to be the existing biological mechanism of backpropagation (Fitzsimonds et al., 1997). Our study applied the backpropagation mechanism to the SNN algorithm and constructed the novel SBP-SNN algorithm (Zhang et al., 2021), significantly reducing the calculation error and improving the model's prediction accuracy. Although the temporal resolution of EEG technique is high, its spatial resolution is limited. Therefore, it is necessary to take the spatial axis into the research of EEG. The method “Neurocube” provided the idea of the fusion of temporal and spatial information (Behrenbeck et al., 2019). Combining temporal–spatial information fusion and self-backpropagation would improve the calculation accuracy and spatial resolution of the SNN.

The emotion generation mechanism results from a dynamic combination of multiple brain areas. The research of fMRI also showed that many brain areas were activated during emotion generation, including the amygdala, lateral prefrontal cortex, ventrolateral prefrontal cortex, superior temporal gyrus, angular gyrus, anterior midcingulate cortex, etc. (Liu et al., 2022; Malezieux et al., 2023). Meanwhile, it is hard to precisely locate the spatial position of the intervention target of transcranial electrical stimulation (tES) and determine the parameters of the intervention signal. Therefore, we assume that there is also an emotional brain network mechanism based on EEG that can be used for emotional decoding, using the self-backpropagation spiking neural network with temporal and spatial compatibility to determine the emotional brain networks and the biomarker of different emotional brain networks. It is of great significance for both the diagnosis of mental disease and the brain–computer interface of neuroregulation.

## Materials and methods

### Subjects

Forty-nine subjects (24 males and 25 females) were recruited from Peking University, China. The average age was 23.00 ± 2.30 years. One participant was excluded due to excessive head movements. The Self-Rating Anxiety Scale (SAS) and Beck Depression Inventory (BDI) were used to screen subjects with anxiety and depression symptoms. All participants were right-handed, had a normal or corrected-to-normal vision, and had no past neurological or psychiatric history. They provided informed consent following the protocol approved by Biomedical Ethics Committee, Peking University.

### Stimuli

**Figure 1:**
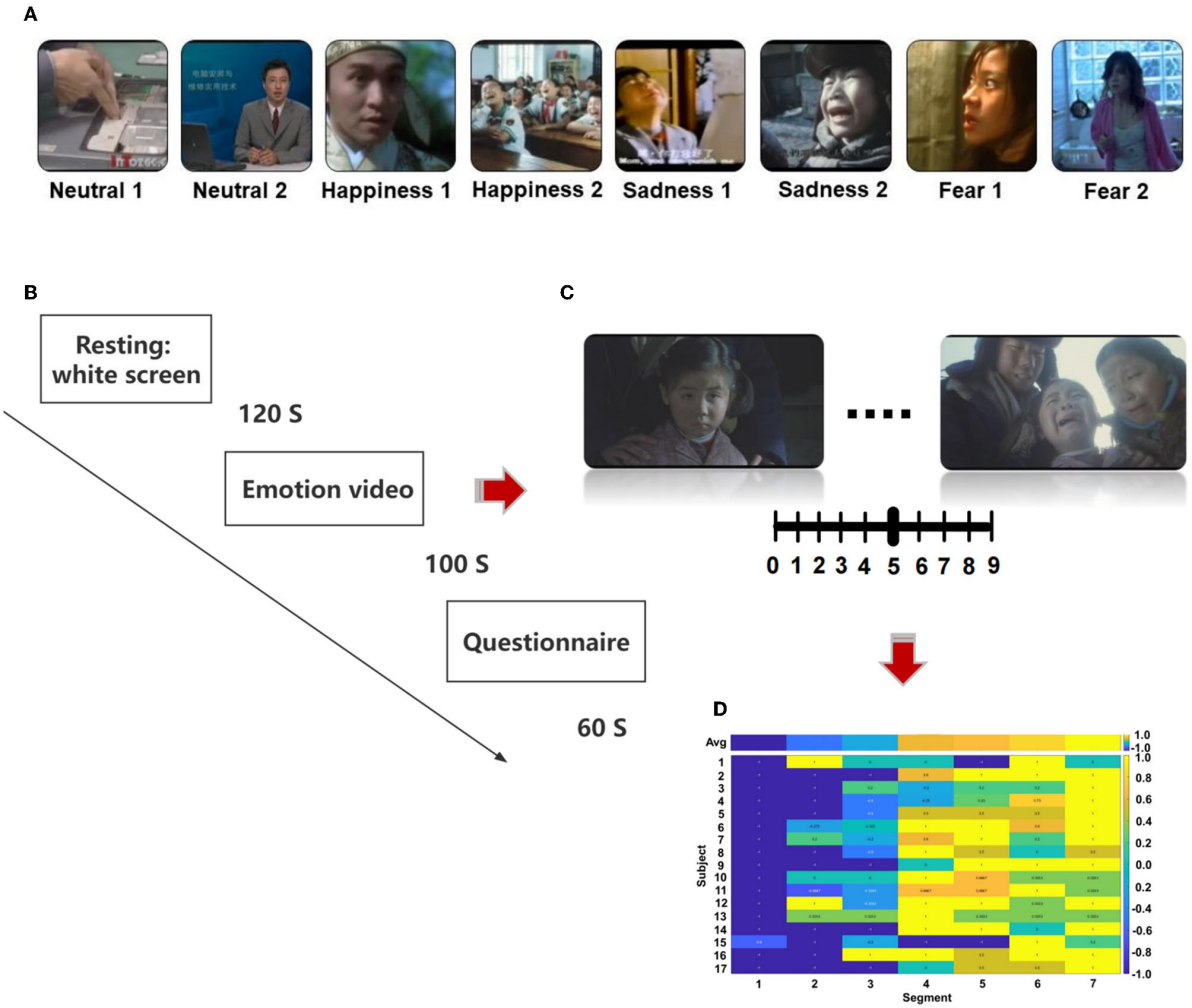
The protocol of the designed emotion experiment, **(A)** sequence of emotional videos, **(B)** process of emotional video clips presentation, **(C)** scoring of emotional vide **(D)** calculation of scoring.

We selected the stimulation from the Chinese Emotional Video System (CEVS). The CEVS is a normalized and localized audiovisual stimulation material library. These videos consist of three basic emotions (i.e., fear, sadness, and happiness) and neutral emotion. For each emotion, we selected two clips. In total, eight clips were used in the research (Figure 1A). A blank screen was shown for 120 s at the beginning of the paradigm, and the video was then played, each lasting 71–247 s. Subjects spent 60 s to finish the questionnaire after the clip finished. The playing sequence was ordered as neutrality 1, neutrality 2, happiness 1, happiness 2, sadness 1, sadness 2, fear 1, and fear 2 (Figure 1B). As shown in Figures 1C, D, every video was scored every 20 s during the playing, with the range of [1, 2, …, 9]. Then, we conducted a cross-subject analysis of the dynamic evaluation during the process of video playing. Video stimulation includes sound and pictures and is more vivid compared with a single sound, picture, and slide. It is similar to the actual scene in life, which is more conducive to the dynamic recognition of emotion. EEG was recorded using BrainVision Recorder (Brain Products, Germany http://www.brainproducts.com/) at a sampling rate of 1,000 Hz from a 62-channel active AgCl electrode cap according to the international 10–20 system. The layout of EEG electrodes on the cap is shown in Supplementary Figure S1. The impedance of each electrode was lower than 10 kΩ. A dimly lit, sound-attenuated, and electrically shielded chamber was used for EEG recording. During the presentation of clips, the subjects were asked to evaluate their feelings every 20 s. For example, how scary the video was, the feeling was scored from 0 to 9. The dynamic changes in emotion were recorded during the video-playing process. Subjects were asked to complete the emotional self-assessment questionnaire with 10 grades at the end of each clip. First, subjects evaluated the intensity of happiness, sadness, fear, and other emotions immediately after watching the clip (0 = no such emotion, 5 = moderate, 10 = extremely strong emotion). Then, the subjects were asked to evaluate the extent of arousal (0 = extremely calm, 5 = no influence, 10 = extremely excited), pleasure (0 = extremely unpleasant, 5 = no influence, 10 = extremely pleasant), dominance (0 = no sense of control, 5 = no influence, 10 = extremely strong sense of control), liking (0 = extremely disgusted, 5 = neither disgusted nor liked, 10 = extremely like), and familiarity (0 = completely unfamiliar, 5 = neither unfamiliar nor familiar, 10 = very familiar).

### Spatial subnetwork division

According to the electrode spatial distribution and the Harvard-Oxford atlas (Desikan et al., 2006), the scalp brain area was divided into seven brain subnetworks as shown in Supplementary Figure S1. Subnetwork 1 mainly covers the prefrontal and frontal areas (Fp and AF). Subnetwork 2 mainly covers the frontal lobe area (F). Subnetwork 3 mainly covers the frontal-central area (FC) and a small frontal-temporal area (FT). Subnetwork 4 mainly covers the central area and part of the temporal lobe area (C and T). Subnetwork 5 mainly covers central-parietal and temporal-parietal areas (CP and TP). Subnetwork 6 mainly covers the parietal brain area (P). Subnetwork 7 mainly covers the parietal-occipital and occipital areas (Po and O).

### Establishment of emotional brain network and discovery of biomarkers

**Figure 2:**
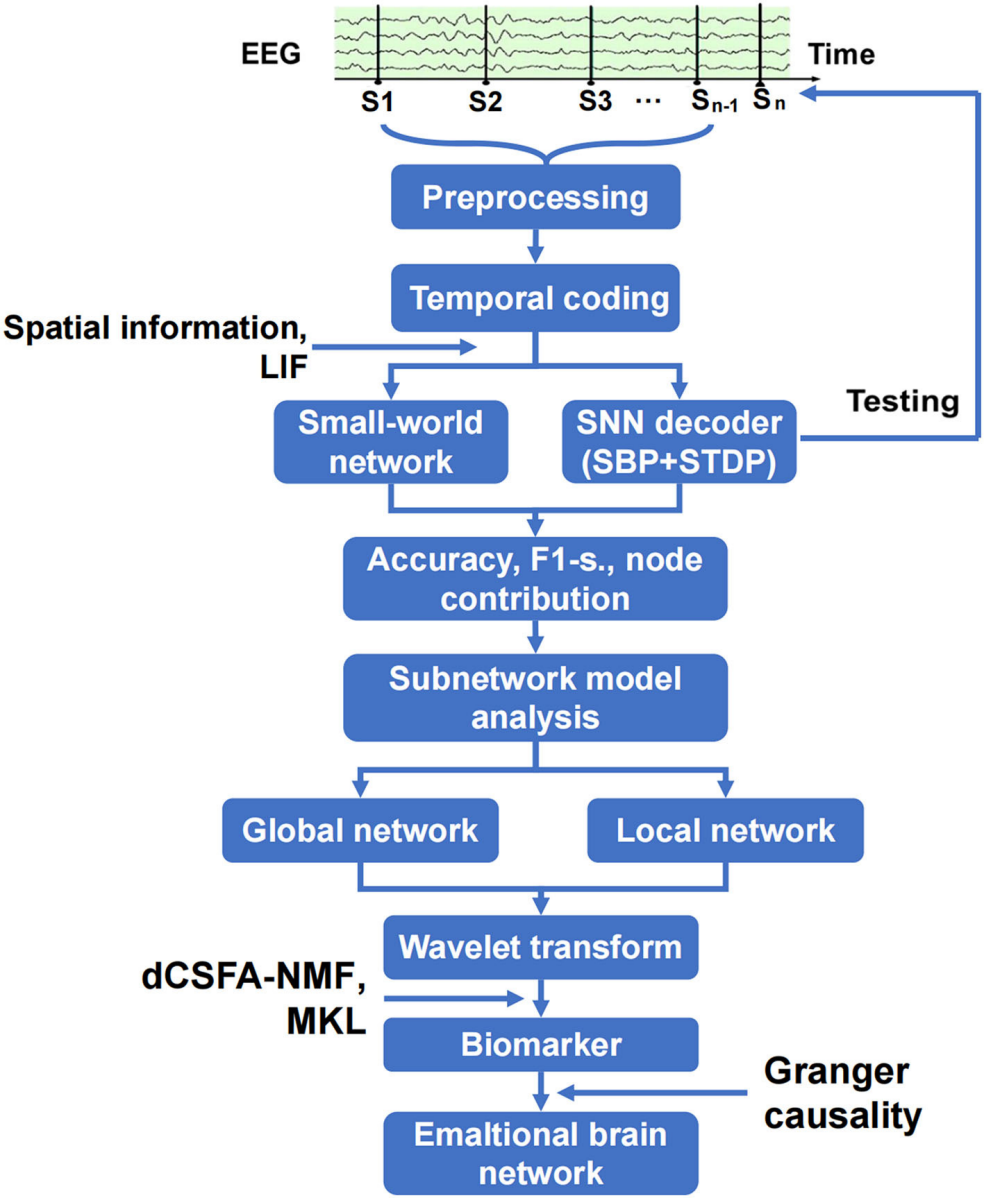
Schematic of spatiotemporal SBP-SNN model used to discover best emotional brain network and biomarker.

The analysis of the emotional brain network is shown in Figure 2. The raw EEG data were cleaned and preprocessed to remove the artifact of eye movement and 50 Hz noise and match the requirements on matrix structure and quality of encoder establishment. The three-layer architecture of SNN, in which SBP and local plasticity (short-term plasticity (STP), spike timing-dependent plasticity (STDP), and homeostatic adjustments of membrane potential adjustment) were introduced at synapses in hidden and output layers, and the teaching spike train was given to the output leaky integrate-and-fire (LIF) neurons. The spatial–temporal SBP-SNN with the spatial coordinates was initialized first, and then, the emotional decoder of SBP-SNN was trained. The accuracy of two indexes and F1-score were adopted to evaluate the performance of the decoder. The small world network algorithm was used to calculate the connection and node weight among all the electrode nodes. The wavelet transform was used to convert EEG data from the time domain to the frequency domain. Multi-kernel learning and discriminative cross-spectral factor analysis non-negative matrix factorization (dCSFA-NMF) were used to discover the biological marker of emotion-related neural oscillation. The directional connection was established using Granger causality method, and then, the emotional brain pattern was obtained.

### Preprocessing

EEG data were preprocessed using MATLAB. The epochs were from−500 ms before the video onset to the video offset. We used a Butterworth bandpass filter from 0.5 Hz to 48 Hz to attenuate power-line noise. Independent component analysis (ICA) was also used to remove artifacts such as eye movements. The Infomax ICA algorithm was used for separating the original signal into independent components. If there were waveforms between these components that were characteristic of eye movements, we removed these eye movements from the signal. Blinks were removed for all participants, and left–right eye movements were removed whenever present (in approximately 50% of the participants). We re-referenced the data by subtracting the average of all the collected electrodes from every single electrode. The baseline correction was performed based on 500ms data before the video onset. Wavelet transform was used to decompose the time-domain EEG data and then obtained the power of different frequency bands in the time-frequency domain, δ (0.5–4 Hz), θ (4–8 Hz), α (8–12 Hz), β (12–30 Hz), and γ (30–48 Hz).

### Spatiotemporal self-backpropagation spiking neural network

According to the research of Zhang et al. (2021), to introduce the SBP mechanism and to provide a simpler demonstration of its impact on SNN, we used a three-layer SNN. In the input layer, neurons received spike trains as inputs encoded using a threshold-based method. The hidden layer consisted of both excitatory and inhibitory leaky integrate-and-fire neurons that exhibited the refractory period, non-linear integration, and non-differentiable membrane potential. The output layer consisted of excitatory LIF neurons that received spiking signals from hidden layer neurons, and the supervised teaching signals were presented only in training procedures. The learning process used both local forms of synaptic modification, that is, STP (Tsodyks et al., 1998; Zucker and Regehr, 2002) and STDP (Diehl and Cook, 2015). When STDP is induced at some specific output synapses, the synaptic weight adjustment will backpropagate with different proportions of long-term potentiation and long-term depression to produce weight adjustment at hidden layer synapses. The restricted Boltzmann machine network (Scellier and Bengio, 2017) was used to examine the effect of introducing SBP into SNN.

According to the research of Kasabov (Kasabov, 2014), 471 nods (coordinate point) were introduced into the spatiotemporal self-backpropagation spiking neural network framework and the coordinates of these neurons correspond directly to the Talairach template coordinates with a resolution of 1 cm^3^. After preprocessing in different subnetwork models, the EEG data were used as the input for emotional decoder training. The label was identified according to video stimuli. Because the EEG signals are time series data with rich time information, the threshold-based encoding method is used for binary temporal spiking coding. The spatial coordinates of spatial nodes and related electrodes are loaded, and the leaky integrate-and-fire neuron and the small-world network are used to initialize the brain spatial model. The SBP-SNN algorithm was used to train the emotional decoder. The number of neurons in the input layer was 100, the number of neurons in the hidden layer was 200, and the number of neurons in the output layer was 1 (for two-class classification) or 4 (for four-class classification). The number of training epochs was 100. The excitatory to inhibitory hidden neurons ratio was 1:1, and the batch size was 20.

### Loss function and synaptic weight

The loss function of SBP-SNN is defined as the standard mean square error, shown as follows:


$$\begin{array}{l} C\;=\;\frac{1}{2}\overset{K}{\underset{k\;=\;1}{\sum }}{\left(u_{k}-o_{k}\right)}^{2} \end{array}$$

where cost is the difference in output *u*_*k*_ and *o*_*k*_ is the expected teaching output. For the RBM using pure backpropagation, the synaptic weight adjustment $W_{j,k}^{BP}$ and $W_{i,j}^{BP}$ can be calculated by the differential chain rule as follows:


$$\begin{array}{l} W_{j,k}^{BP}\;=\;-\eta \frac{\partial C}{\partial W_{j,k}}\\ W_{i,j}^{BP}\;=\;-\eta \frac{\partial C}{\partial W_{i,j}} \end{array}$$

where η is the learning rate, and *i*, *j*, and *k* represent the indices of neurons in input, hidden, and output layers, respectively.

### Cluster analysis

To establish the performance clusters of best fusion models (BFM) and simplest fusion models (SFM), non-supervised K-means cluster analysis was used, setting the number of segments (k) at 2. Each segment is randomly given a center point. The distance is calculated between the center point and every accuracy and F1-score. Then, the points nearing the center point are divided into clusters, and every new cluster's new center point can be calculated. Iterating the process above until the center point remains unchanged by K-means.

### Connectivity

Functional brain connectivity manifests the small-world organization across different time scales (Bullmore and Sporns, 2009). Neurons in a structural or functional area of the brain are more densely interconnected, and the closer these areas are, the higher the connectivity between them. Therefore, the electrode connection was calculated using the small-world connectivity algorithm (Watts and Strogatz, 1998), where a radius is defined as a parameter for connecting electrodes within this radius, with small weight values attached to the connections which are 80% positive. Long-distance connectivity could be used to calculate connections beyond the radius of small-world connectivity. Connection weight is the electrode weight between a pair of electrodes. It is adjusted during unsupervised learning to reflect the interaction between the electrodes.

### Granger coherence

The spectral Granger causality (Geweke, 1982) features were calculated using the MATLAB-based Multivariate Granger Causality (MVGC) toolbox (Barnett and Seth, 2014). Granger causality values for each window were calculated using a 20-order AR model via the GCCA_tsdata_to_smvgc function. Granger causality values were calculated for all integer frequency values within the desired range for all directed pairs of electrodes in the dataset (Mague et al., 2022).

### Multiple kernel learning analyses of distinct frequencies

To comprehensively assess the possible contributions of different frequency bands of activity to task-evoked EEG responses, we performed a multiple kernel learning (MKL)-based analysis. The MKL approach is a machine learning-based method for feature selection that can be applied to classifying EEG task conditions by including multiple frequency bands of activity as well as multiple electrodes in a single model (Kucyi et al., 2020). In addition, easy MKL was used to classify emotion categories (Aiolli and Donini, 2015).

## Results

### Evaluation of emotional stimulation paradigm

The properties of dynamic visual and audio stimuli make movies one of the most effective ways to elicit emotions (Wang et al., 2014). After eight videos, subjects scored valence, arousal, dominance, liking, and familiarity. The affect intensity and hit rate of six non-neutral videos were calculated. All the results are shown in Supplementary Table S1. The affect intensity of all the non-neutral videos is above 5 (medium strength). The hit rate of the three emotions is over 90%. The results indicate that the chosen videos are of good validity, which could effectively elicit related emotions.

### Difference analysis between emotional and neutral stimulation

**Figure 3:**
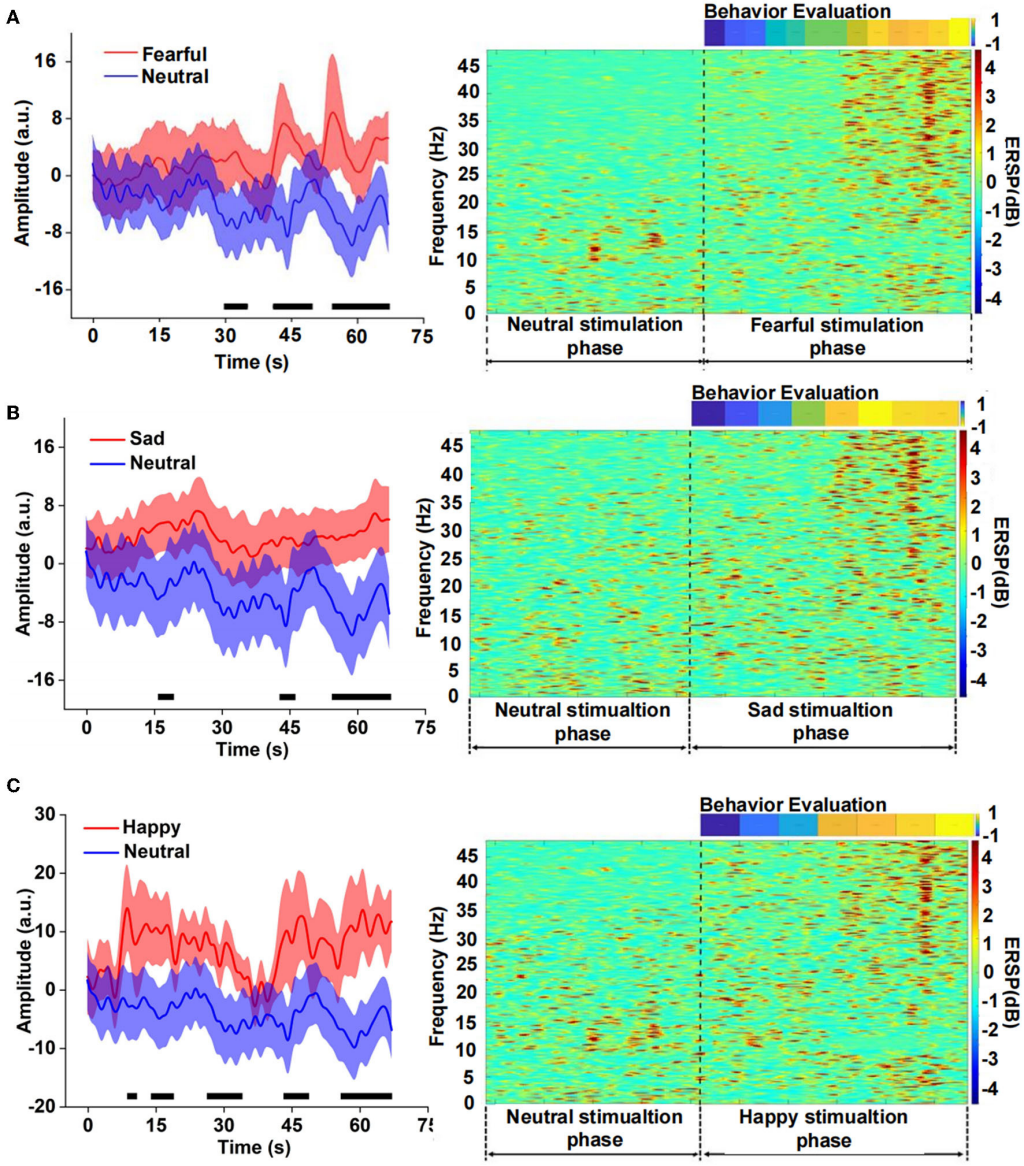
Comparison of the neutral amplitude changes in a time domain and event-related spectral perturbation (ERSP) in the time-frequency domain between different emotions, **(A)** fear versus neutrality, **(B)** sadness versus neutrality, and **(C)** happiness versus neutrality.

We compared the differences between fear and neutral emotion-related EEG in the time domain and found that the EEG amplitude of fear was significantly higher than that of neutral after 30 s (Figure 3). There was also a significant difference between sad and neutral stimuli, the amplitude of the latter was much lower than the former, mainly in the late stage of the clip presentation. The emotion of happiness has similar results to fear and sadness, and there was a significant difference in the EEG amplitude between happiness and neutrality, especially in the middle and late stages. Event-related spectral perturbation (ERSP) of different emotions further indicated difference in the power between fearful and neutral conditions. The former is significantly higher than the latter, especially the frequency band (15–48) Hz during the late stage of the clip. There were similar results for the sad and neutral emotions. However, the power of ERSP for happiness was significantly inhibited in the frequency range of (8–15) Hz, which still occurred in the middle and late stages, according to the analysis of the amplitude and power of different emotions EEG in the time and time-frequency domains above. It could be seen that the apparent difference mainly occurred in the middle and late stages of the video playback, which indicated that there was an energy difference between the emotional component and the neutral component of the video stimulation. Meanwhile, the emotional expression had a cumulative effect over time.

### Emotional subnetwork model analysis

**Figure 4:**
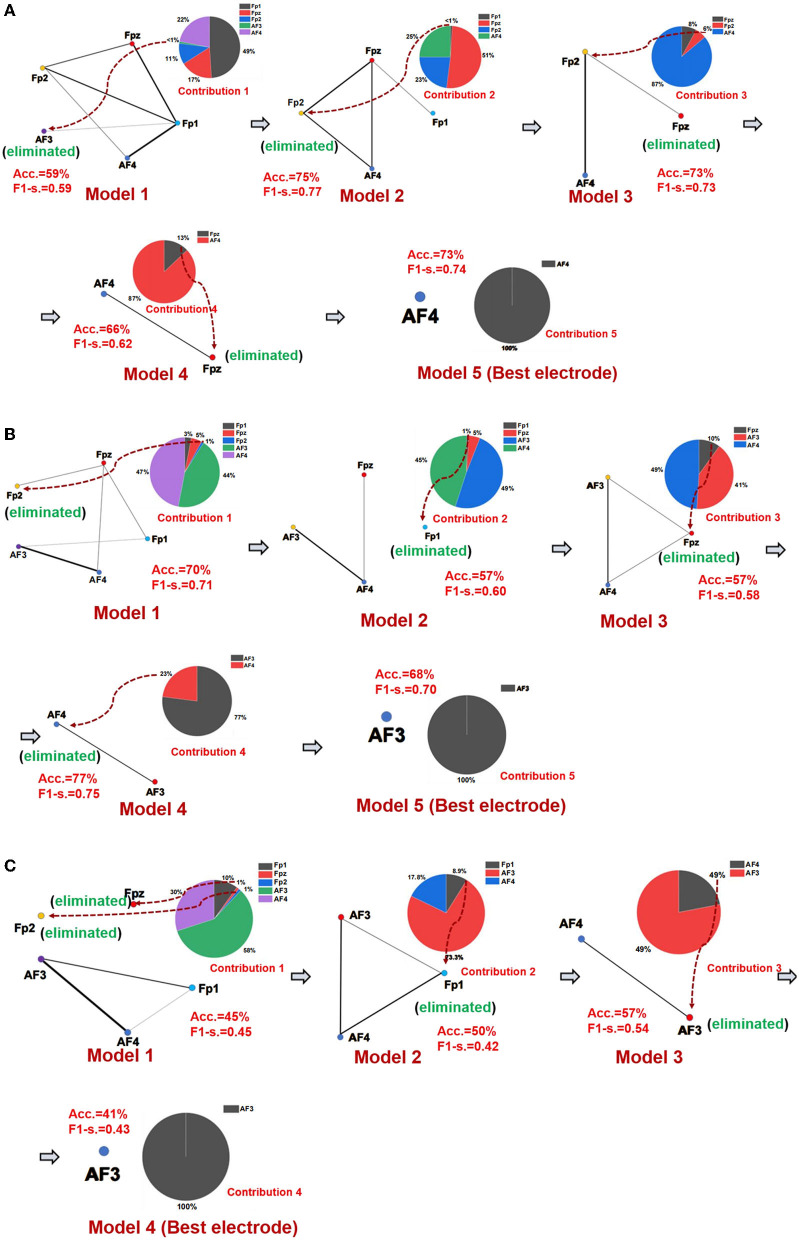
Emotional brain subnetwork model and its optimization process. **(A)** The first model mining process of fear, **(B)** the first model mining process of sadness, and **(C)** the first model mining process of happiness.

The best emotional network model and the best electrode location of seven networks of three emotions were established using the SBP-SNN. First, as shown in Figure 4, the initial network model and the contribution of every node in the model were calculated. Next, the node with the lowest contribution was eliminated according to prediction accuracy and F1-score to generate a new subnetwork model, and then, the performance of the new model and the participation of each electrode node were calculated. This process would continue until the emotional subnetwork model only had the last node or the prediction model performance continued to be poor; that is, the brain area covered by the relevant electrode did not participate in the arousal of the corresponding emotion.

As shown in Figure 4A, in the first subnetwork of fearful emotion, the electrodes of the initial network at the frontal area include Fp1, Fpz, Fp2, AF3, and AF4, in which Fp1 had the largest contribution in the whole model, forming an initial subnetwork model with Fp1 as the core node, Fpz, AF3, Fp2, and AF4 electrodes were also involved. In the initial subnetwork model of the first subnetwork, the accuracy and F1-score were 59% and 0.59, respectively. The contribution of the AF3 electrode was the smallest, even lower than 1%. Therefore, AF3 would be eliminated in the next model. According to the principle of minimum contribution electrode elimination in emotional subnetwork mining, AF3, Fp1, Fp2, and Fpz were eliminated in the continuous optimization of the models, and the simplest subnetwork with an AF4 node was finally determined. The prediction accuracy and F1-score were 73% and 0.74 in the simplest subnetwork model. The second model (Fpz, Fp2, AF4, Fp1) had the best performance in the fear first subnetwork model, the accuracy was 73%, and the F1-score was 0.74. The optimization process of the other six subnetwork models is similar to the first subnetwork. The whole performances of all fear subnetworks models are shown in Supplementary Table S2.

It should be noted that Fp1 was the core electrode in the initial subnetwork model of the first subnetwork of fear. However, the Fp1 electrode was eliminated in the establishment process of the third subnetwork model, and it did not finally become the best electrode, which showed that rather than independently, each electrode played a role as a part of the emotional brain network model. As we know, emotion generation in the local brain area usually depends on the whole brain network. Therefore, structural changes in a specific emotional brain network might correspond to the overall emotion. From the perspective of the electrode site, the node that performed best in one of the brain networks might not be the one that best reflects emotion. The mining process of the sadness first subnetwork is shown in Figure 4B, and the core electrode of the initial subnetwork model was AF4. Fp2, Fpz, Fp1, and AF3 electrodes were also involved. The accuracy and the F1-score of the initial emotional subnetwork model were 70% and 0.71, respectively. After a series of model optimizations and the electrode with worst-performance elimination, model 4 achieved the best performance, with an accuracy of 77%, and the F1-score of 0.75. The core electrode of the simplest subnetwork model was AF3, the accuracy was 68%, and the F1-score was 0.70 in the model. Similarly, the simplest subnetwork model electrode was not the best electrode in the initial subnetwork model. The analysis process of other subnetworks of sadness is similar to the first subnetwork, and the model performance of all subnetworks is shown in Supplementary Table S3. The happiness first subnetwork optimization process is shown in Figure 4C. The initial subnetwork model was composed of Fp2, Fpz, Fp1, AF3, and AF4, where AF3 was the core electrode of the model. The model's prediction accuracy was 45%, and F1-score was 0.45. The best model was model 3 after the optimization, the accuracy was 57%, and the F1-score was 0.54. The electrode of the simplest subnetwork model was AF3, the same as the core electrode of the initial subnetwork. The other subnetworks of happiness mining processing are similar to the first subnetwork. The performances of all happiness prediction models are shown in Supplementary Table S4.

As shown in Supplementary Table S2, model 2 had the best performance among all models in subnetwork 1 of fearful emotion. The simplest model was model 4. The best model was model 1, and the most straightforward model was model 4 in subnetwork 3. Model 2 had the best performance, and model 4 was the simplest in the subnetwork 4. In subnetwork 5, model 4 was the best, and the most straightforward model was model 5. Model 3 was the best, and model 7 was the simplest in subnetwork 6. In subnetwork 7, model 3 had the best performance; the most straightforward model was model 7. Comparing all these models, we found that model 3 in subnetwork 6 had the best performance among all the best models. The accuracy of model 3 was 84.09%, and the F1-score was 0.84. All performances of sadness subnetwork models are shown in Supplementary Table S3. Model 3 from subnetwork 6 had the best performance among all the best models. The prediction accuracy was 84.09%, and the F1-score was 0.86, which were much higher than other models. Supplementary Table S4 shows all results of the happiness prediction models of seven subnetworks. There was the same performance between model 3 of subnetwork 3 and model 1 of subnetwork 4. The prediction accuracy was 81.82%, and the F1-score was 0.83. However, according to the subnetwork mining process, especially the performance of the simplest models of the two models, the overall performance of subnetwork 3 was better than that of subnetwork 4.

### Emotional subnetwork models fusion analysis

**Figure 5:**
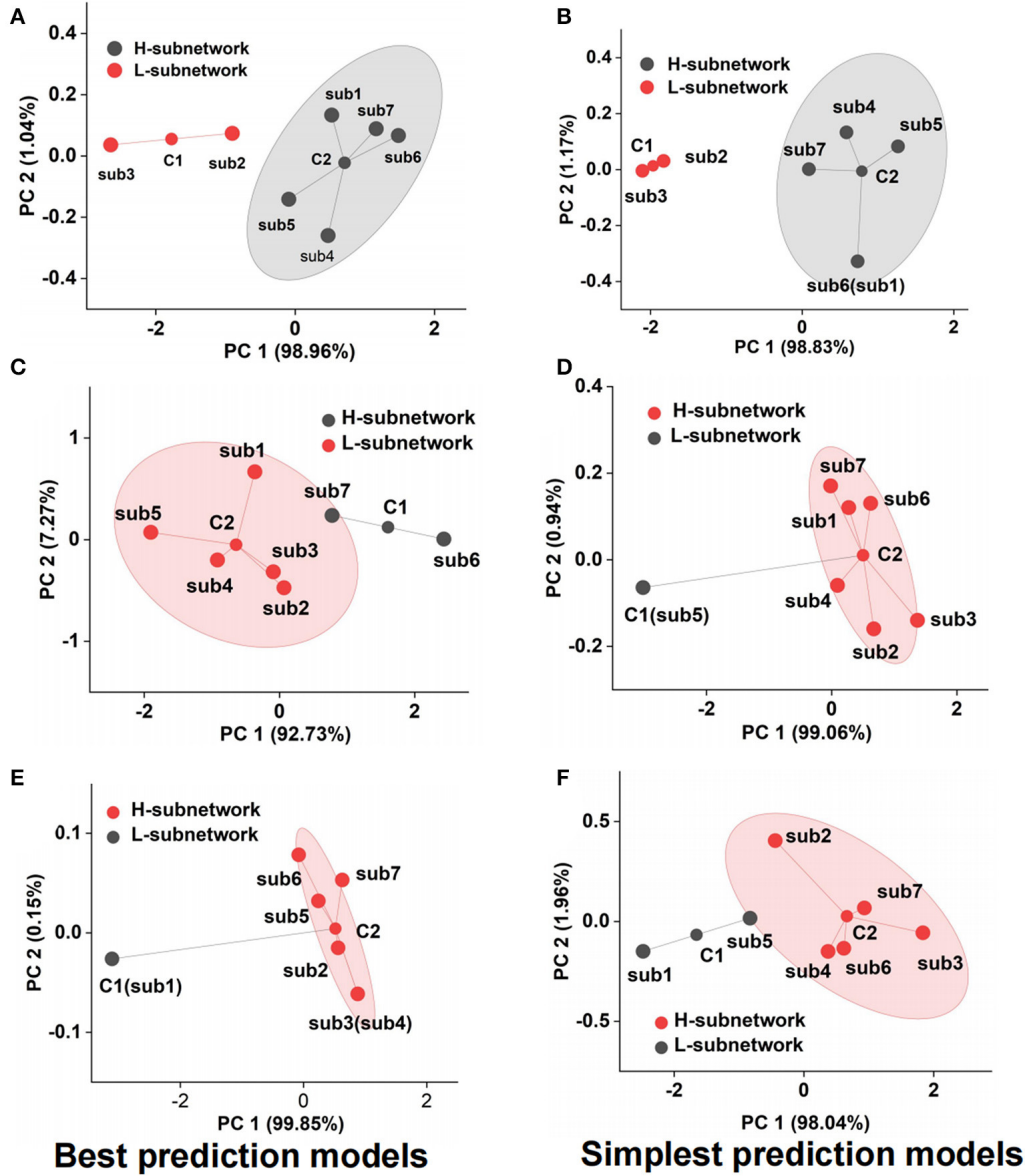
Performance cluster of subnetwork model, **(A)** fear best model cluster, **(B)** fear simplest model cluster, **(C)** sadness best model cluster, (**D)** sadness simplest model cluster, **(E)** happiness best model cluster, and **(F)** happiness simplest model cluster.

Although we adopted the accuracy and F1-score to assess the emotional subnetwork model's performance quantitatively, it is difficult to discriminate whether there were differences among seven subnetworks on the best and simplest models from the indicators. Therefore, cluster analysis provided the possibility for model fusion across subnetworks. As shown in Figure 5A, the best model performance of subnetwork 2 and subnetwork 3 was not the same class as that of subnetwork 1,4, 5, 6, and 7 by cluster analysis. The latter's performance was much better than the former's, which meant that the best models from the subnetwork 1, 4, 5, 6, and 7 could better reflect the fearful emotion. The cluster results on the simplest models showed that the model performance of subnetworks 1, 4, 5, 6, and 7 was better than that of subnetworks 2 and 3 in the fear prediction (Figure 5B). The best model in subnetwork 6 differed from the other six subnetworks in sad emotion (Figure 5C). As shown in Supplementary Table S3, the performance of the best model of subnetwork 6 was the best compared with the other six best models. The prediction accuracy was 84.09%, and the F1-score was 0.86. The performance of subnetwork 5 was worse than the other six subnetworks on the simplest model (Figure 5D). The prediction accuracy of the model was 47.73%, and F1-score was 0.41. In the analysis of clustering of subnetwork models of the emotion of happiness, the performance of the best model of subnetwork 1 was significantly different from the other six subnetworks (Figure 5E). Subnetwork 1 differed from the other six subnetworks in the simplest subnetwork (Figure 5F). Considering the specific indexes in Supplementary Table S4, the performance of the other six subnetwork models was significantly better than that of subnetwork 1. The prediction accuracy was 56.82%; F1-score was 0.56. According to the clustering results above, several networks could be clustered and may contribute together to emotion generation. Therefore, it is necessary to explore the role of fusion models from the best model fusion and the simplest model fusion. Meanwhile, the performance was compared on best BFM, SFM, and signal best subnetwork models (BSM) to determine which was the best structure of the emotional brain network. The basic structure of fear best fusion model included the best models in subnetworks 1, 4, 5, 6, and 7. The subnetwork component of the simplest fusion model was the same as the best fusion model. All seven subnetworks were included in the best fusion model for sadness model fusion. Subnetworks 1, 2, 3, 4, 6, and 7 were included in the sad simplest fusion model. The best fusion model of happy included subnetworks 2, 3, 4, 5, 6, and 7. The structure was the same as that of the simplest fusion model. Therefore, we used multi-subnetworks EEG data as the input of the SBP-SNN to establish emotion evaluation models.

As shown in Table 1, the prediction accuracy of the BFM was 70.45%, and F1-score was 0.74. The prediction accuracy of the SFM was 65.91%, and F1-score was 0.67. However, the prediction accuracy of the BSM among all the subnetworks was 81.82%, and the F1-score was 0.84. The prediction accuracy of the BFM of sadness was 72.23%, and the F1-score was 0.76. The prediction accuracy of the SFM of sadness was 70.45%, and the F1-score was 0.73. The prediction accuracy of the BSM among all models in Supplementary Table S3 was 84.09%, and F1-score was 0.86. The prediction accuracy of the BFM of happiness was 79.55%, and the F1-score was 0.84. The prediction accuracy of the SFM was 77.72%, and the F1-score was 0.78. However, the best model among all the subnetwork models in Supplementary Table S4 had the best performance, and the accuracy and the F1-score were 81.82% and 0.83, respectively. According to the results above, the performance of BFM was better than that of the SFM but worse than that of the BSM among all subnetworks for all three emotions.

**Table 1:** Comparison of the emotion prediction performance of BFM, SFM, and BSM.

**Index**	**Fear**			**Sadness**			**Happiness**		
	**BFM**	**SFM**	**BSM**	**BFM**	**SFM**	**BSM**	**BFM**	**SFM**	**BSM**
Acc. (%)	70.45	65.91	81.82	72.73	70.45	84.09	79.55	77.27	81.82
F1-Sc.	0.74	0.67	0.84	0.76	0.73	0.86	0.82	0.78	0.83

**Figure 6:**
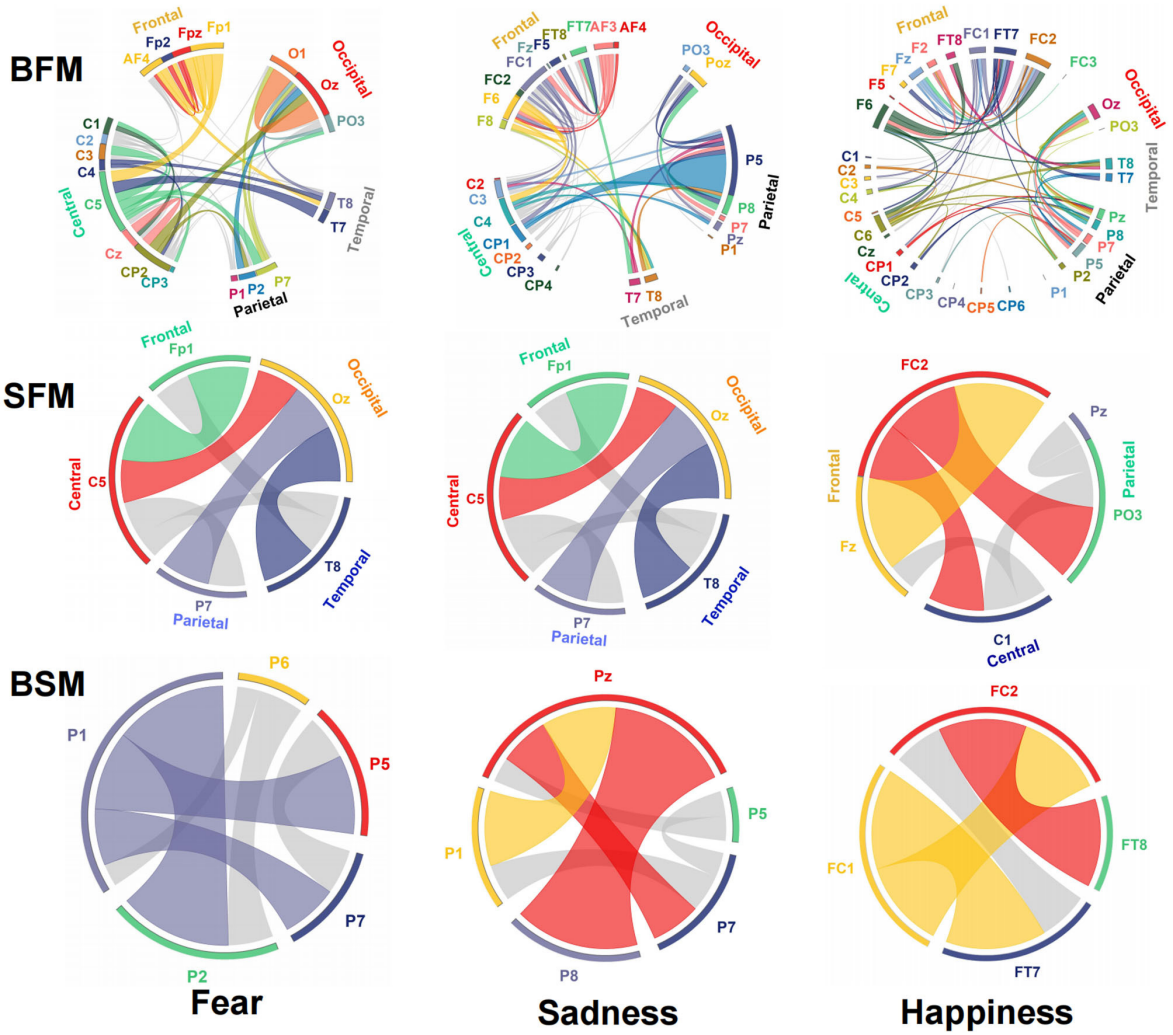
Structure and functional connectivity of emotional brain network model, BFM, SFM, and BSM.

The emotional EEG brain network is shown in Figure 6. There were 20 electrodes in the BFM of fear, the corresponding brain area involved frontal, central, parietal, temporal, and occipital areas. The central area had the largest number of electrodes. The number of SFM electrodes was more streamlined comparing the BFM; it was also distributed in the five brain areas. However, there was a significant decline in the prediction accuracy and F1-score from the BFM to the SFM (Table 1). The BSM of fear is located parietal lobe, and its structure includes P1, P2, P5, P6, and P7. The results of the BFM of sad showed that there 25 electrodes were involved. They were distributed in the frontal, central, temporal, parietal, and occipital areas. The emotional brain area of sadness was related to the frontal-central-occipital lobe in the SFM. Most electrodes were located in the frontal lobe. Meanwhile, with the decrease in the number of electrodes from the BFM to the SFM, the prediction ability also decreased. The BSM of the sadness was located parietal lobe, and its structure includes P1, P5, Pz, P7, and P8. Most electrodes were related to happiness; there were 33 electrode sites activated, involving frontal, central, parietal, temporal, and occipital areas. The number of electrodes from the frontal lobe was the largest. The brain area of the SFM of happiness included the frontal, central, and occipital lobes. The number of electrodes from the frontal area was also the largest. The model's performance became worse with the decrease in the participated electrodes. The BSM of happiness was located in frontal-central and frontal-parietal areas, and its structure includes FC1, FC2, FT7, and FT8.

### Emotional biomarkers and brain network based on the BSM

**Figure 7:**
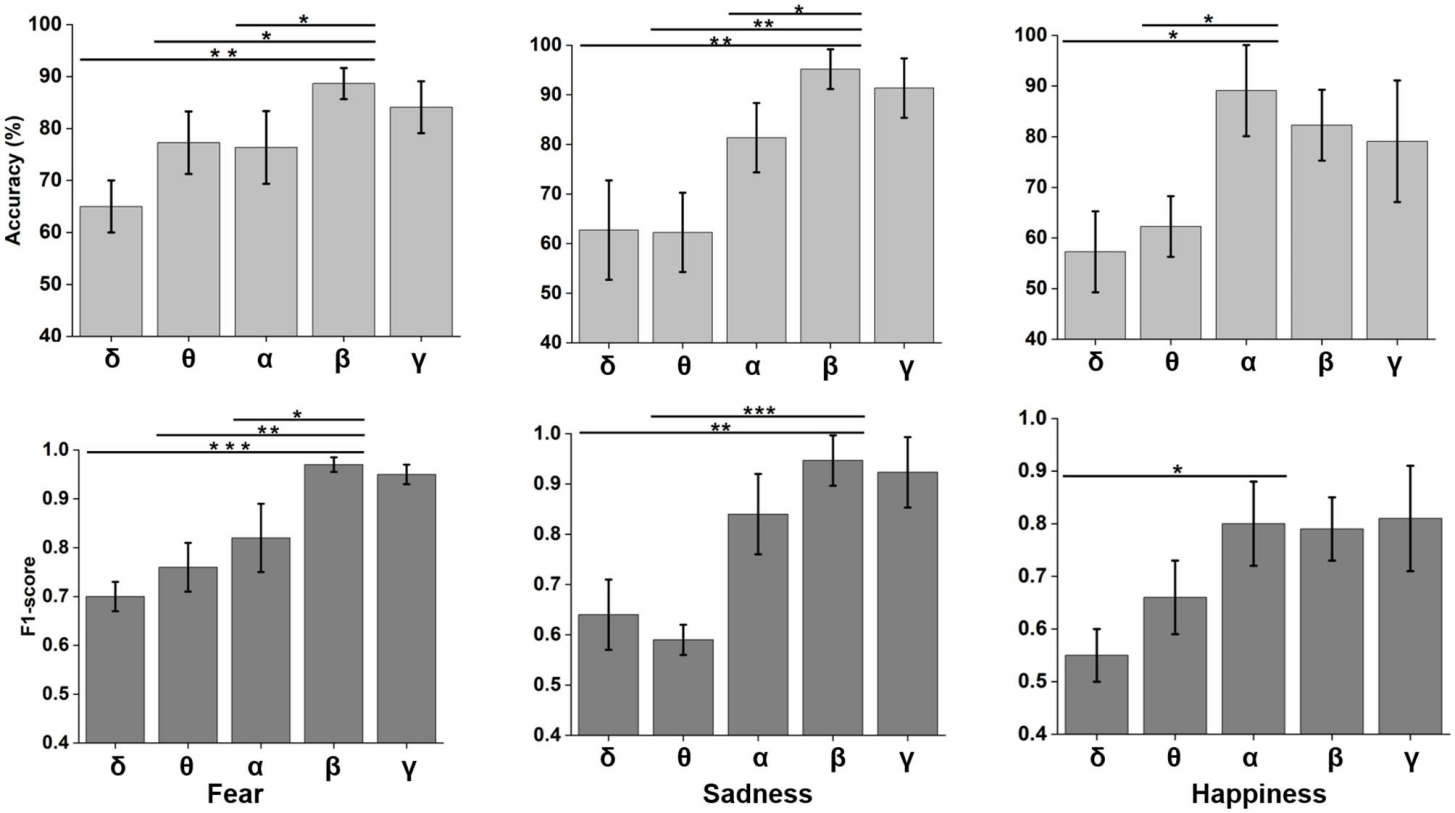
Neural oscillation modeling and prediction of different emotions by the SBP-SNN. Asterisks indicate significant *p* value as *p < 0.05, **p < 0.01 and ***p < 0.001 (two-tailed t-tests). Error bars represent SEMs.

Neural oscillation was an important indicator of brain state. δ (0.5–4 Hz), θ (4–8 Hz), α (8–12 Hz), β (12–30 Hz), and γ (30–48 Hz) frequency bands were decomposed using wavelet transform from time domain emotional EEG data. As shown in Figure 7, the SBP-SNN was used to decode brain activity of emotion. To our knowledge, every frequency band was used in the brain to perform not just one but multiple functions. In turn, each function could be represented by multiple oscillations. In our research, the changes in emotion could be reflected by relatively higher frequency bands (α, β, and γ) for fear, sadness, and happiness. The prediction accuracy of the β frequency band on fear was significantly different from that of the δ, θ, and α frequency bands and higher than that of the γ frequency band. The F1-score of the β band was also higher than that of the other frequency bands. The same results were found in sadness prediction. Although the prediction accuracy of the α frequency band in happiness was significantly different from those of δ and θ frequency bands, the accuracy was also higher than that of β and γ frequency bands. The mean F1-score in α frequency bands was higher than 0.8.

**Figure 8:**
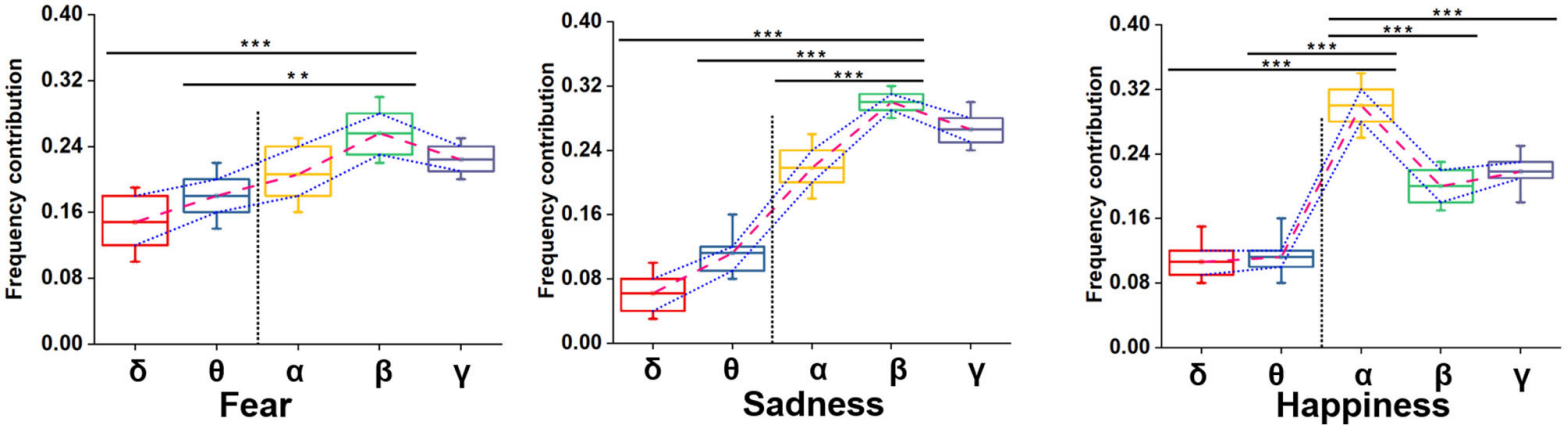
Summed contribution of each frequency band to the full model (averaged across folds) using MKL, in percent. Asterisks indicate significant *p* value as **p < 0.01 and ***p < 0.001 (two- tailed t-tests). Error bars represent SEMs.

We adopted multiple kernel learning (MKL) to decompose the relative contributions of the power amplitudes of distinct frequency bands (Schrouff et al., 2016). Figure 8 shows a significant difference in the β and the lower frequency bands (δ and θ). Meanwhile, the contribution of the β frequency band was the highest for fear and sadness. The contribution of the α frequency band was highest for happiness, and it significantly differed from the other four frequency bands. According to the results above, the β frequency band could be the biological marker of negative emotions: fear and sadness. However, the β frequency band could not be used to distinguish the two emotions internally. α oscillation could be used as a biological marker of happiness.

**Figure 9:**
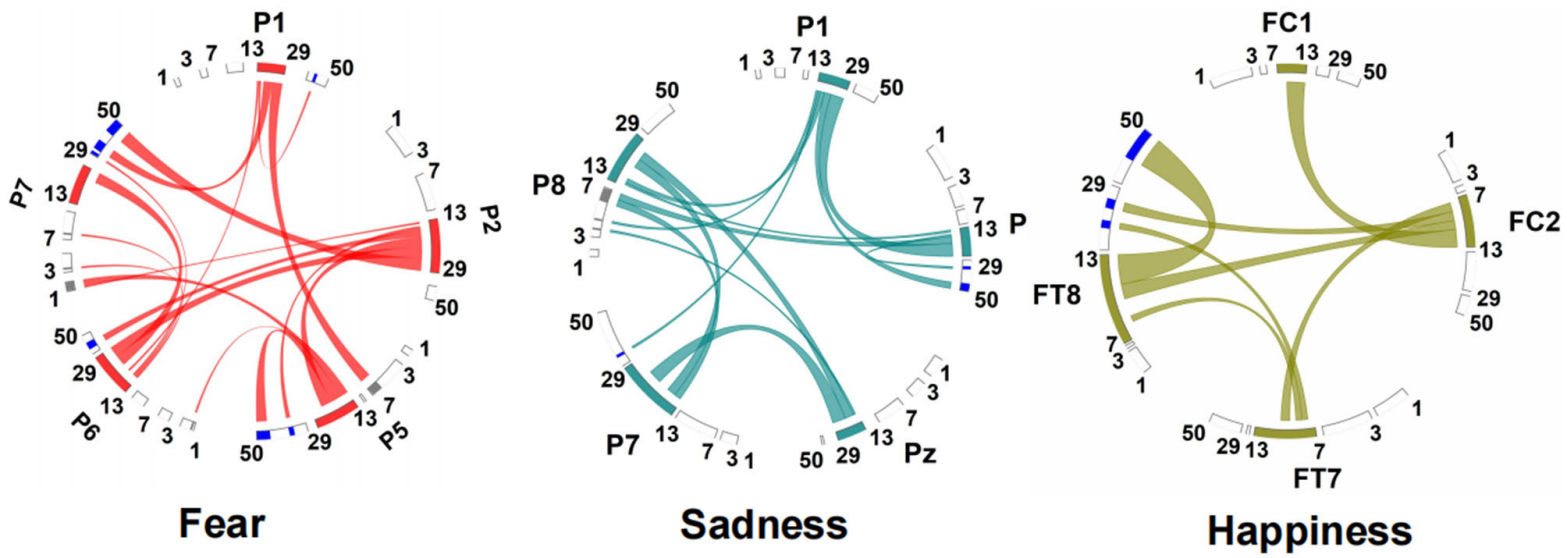
Power and synchrony measures compose BSM of three different emotions. Brain areas and oscillatory frequency bands ranging from 1 to 50 Hz are shown around the rim of the circle plot. The highlights around the rim depict spectral power measures that contribute to the BSM, and cross-spectral (i.e., synchrony) measures are depicted by the lines connecting the brain regions through the center of the circle.

The connection between neural oscillations from different neural network nodes was crucial. The best model of fear was model 3 of subnetwork 6. Parietal electrodes including P1, P2, P5, P6, and P7 were included in the model. Discriminative cross-spectral factor analysis non-negative matrix factorization (dCSFA-NMF) (Talbot et al., 2020) was used to discover the network structure within neural data. As shown in Figure 9, 13–29 Hz (β) oscillation was also characteristic of fear for all five electrode sites. The transmission of β oscillation at different nodes was mainly concentrated in the same frequency band and higher frequency band. The subnetwork model with the best performance of sadness emotion was model 3 of subnetwork 6. The electrode nodes included P1, P5, Pz, P7, and P8, which are also located in the parietal lobe. β (13–29 Hz) frequency band was still the main characteristic of emotion generation in different nodes. The energy connection between different nodes also mainly occurred in the same frequency band, a small part of high-frequency and low-frequency oscillation. The best subnetwork model of happiness was model 3 of subnetwork 3, which included electrode nodes FC1, FC2, FT7, and FT8. These nodes were located in the frontal-central and frontal-temporal lobes. Among the four electrode nodes, α (7-13 Hz) oscillation was essential to happiness generation. The connectivity of neural oscillation between different nodes mainly occurred in the same frequency band (α) and secondly higher frequency bands (β and γ).

**Figure 10:**
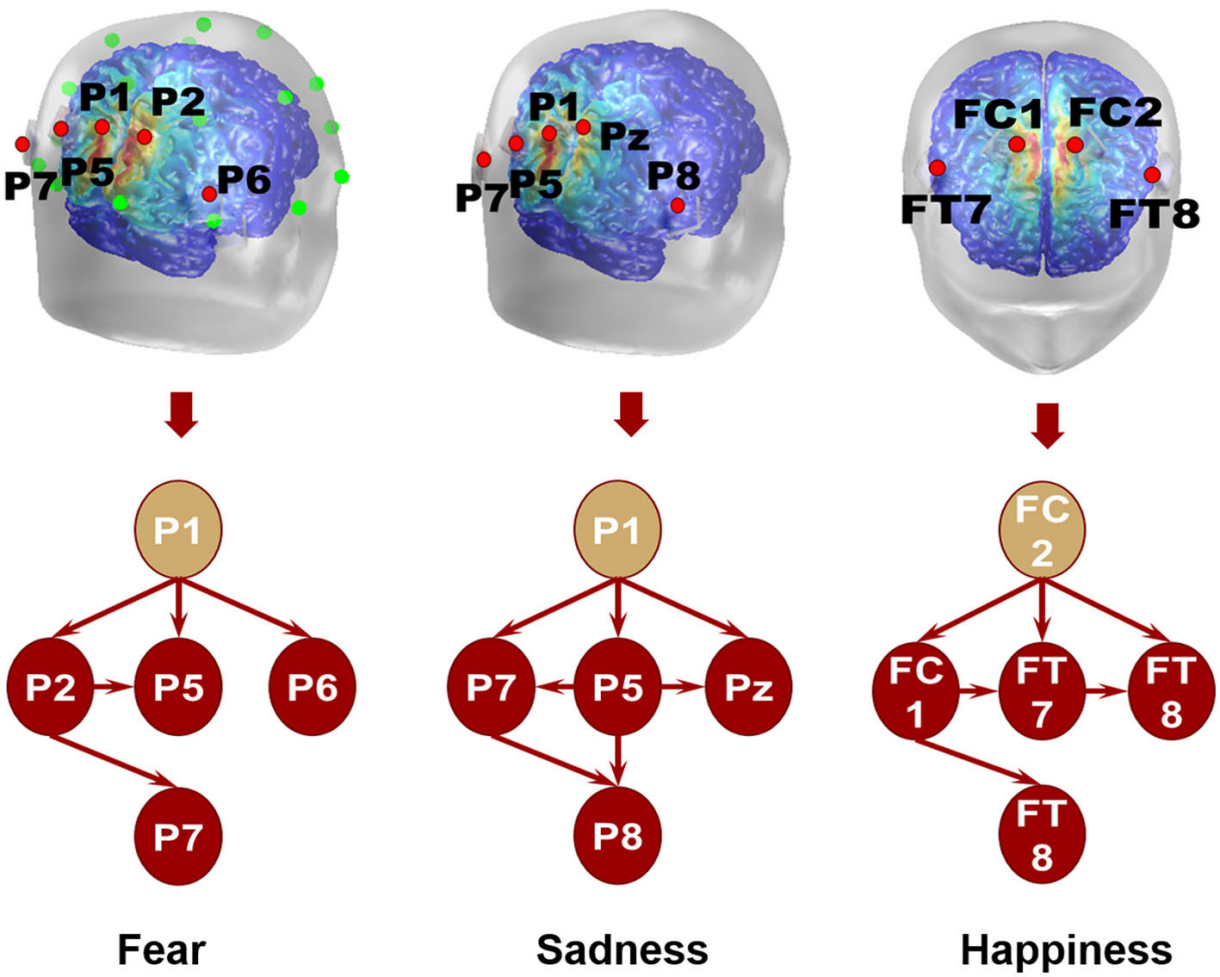
Local brain network of different emotions.

The directional connection of different nodes was an essential part of the connectivity evaluation of the emotional brain network. Granger causality analysis of the fearful emotion generation brain network showed that the projection of β oscillation was from P1 to P2, P5, and P6. There was no prominent projection to P7. Furthermore, there was an oscillation projection from P2 to P5 and P7. There was no causality between other electrode nodes, as shown in Figure 10. The β oscillation from node P1 projected directly to P5, P8, and P7 for sadness. There were direct projections from P5 to Pz, P7, and P8. There were further direct projections from P5 and P7 to P8. For happy emotion, there were direct projections from FC2 to FC1, FT7, and FT8. The oscillation from FC1 projected to FT7 and FT8. Finally, there was a projection from FT7 to FT8. Therefore, P1 and FC2 could be essential intervention targets in emotion regulation research.

### Performance evaluation of emotion decoding algorithms

We next compared the effect of different machine learning algorithms in predicting emotion, including binary classification and four-class classifications as shown in Table 2. The classification accuracy of the SNN algorithm for fear, happiness, and sadness is 76.72%, 75.48%, 78.19%, and 53.88%, respectively. The performance was superior to logistic regression, naive Bayes, and K-neighborhood algorithm and was basically equivalent to support vector machine and random forest algorithm, but far lower than CNN-LSTM fusion algorithm. By introducing the SBP mechanism, the accuracy of emotion decoding of the SBP-SNN algorithm was significantly improved. The prediction accuracy of fear, sadness, and happiness was 81.82%, 84.09%, and 81.82%, respectively, and the accuracy of the four-class classifications was 63.86%. The classification accuracy of the CNN+LSTM algorithm in the above four-class classification predictions was 87.50%, 75.00%, 81.25%, and 61.00%, respectively. The SBP-SNN had advantages in predicting the binary and four-class classification of sadness and happiness, except that the binary classification performance of fear was slightly lower than the CNN+LSTM.

**Table 2:** Emotion prediction performance of machine learning algorithms in the time domain.

**Class**	**Index**	**LR**	**NB**	**SVM**	**RF**	**KNN**	**CNN-LSTM**	**SNN**	**SBP-SNN**
Fearful and Neutral	Acc. (%)	28.62	47.67	71.94	76.24	66.74	87.50	76.72	81.82
	F1-Sc.	0.28	0.55	0.48	0.72	0.58	0.75	0.74	0.84
Sad and Neutral	Acc. (%)	57.14	52.44	66.71	42.96	56.22	75.00	78.19	84.09
	F1-Sc.	0.39	0.39	0.73	0.26	0.52	0.72	0.80	0.86
Happy and Neutral	Acc. (%)	33.33	47.36	72.41	66.75	60.11	81.25	75.48	81.82
	F1-Sc.	0.25	0.25	0.64	0.68	0.58	0.72	0.76	0.83
Four classes	Acc. (%)	–	55.63	62.62	53.37	56.05	61.00	53.88	63.86
	F1-Sc.	–	0.44	0.57	0.43	0.50	0.59	0.56	0.66

As shown in Tables 2, 3, the prediction accuracy and F1-score of binary and four-class classifications for all algorithms were improved in the time-frequency domain compared with the time domain. The prediction accuracy and F1-score of the SBP-SNN algorithm in fear, sadness, happiness, and four categories were 86.36% and 0.92, 95.18% and 0.95, 89.09% and 0.80, 70.25% and 0.73, respectively. The results further proved the reliability and necessity of biomarker identification of β and α frequency bands. Compared with the SNN and other algorithms, the SBP-SNN algorithm retained the spiking efficient coding characteristic of the third-generation neural network and improved the accuracy of SNN.

**Table 3:** Emotion prediction performance of machine learning algorithms in the frequency domain.

**Class**	**Index**	**LR**	**NB**	**SVM**	**RF**	**KNN**	**CNN+LSTM**	**SNN**	**SBP-SNN**
Fearful and Neutral	Acc. (%)	31.48	52.44	79.13	83.86	73.41	89.25	82.18	86.36
	F1-Sc.	0.31	0.61	0.53	0.79	0.64	0.83	0.82	0.92
Sad and Neutral	Acc. (%)	62.85	57.68	73.38	47.26	61.84	82.50	86.24	95.18
	F1-Sc.	0.43	0.43	0.80	0.29	0.57	0.79	0.85	0.95
Happy and Neutral	Acc. (%)	36.66	52.10	79.65	73.43	66.12	89.38	83.56	89.09
	F1-Sc.	0.28	0.28	0.70	0.75	0.64	0.79	0.85	0.80
Four classes	Acc. (%)	–	61.19	68.88	58.71	61.66	67.10	65.35	70.25
	F1-Sc.	–	0.48	0.63	0.47	0.55	0.65	0.70	0.73

## Discussion

As we know, many brain areas are involved in the neural circuit of emotion generation. Meanwhile, the structure of emotional brain networks was very complex. The pathways of neural circuits, such as fear, have been proven to be the ventromedial prefrontal cortex, amygdala, orbitofrontal cortex, insular cortex, nucleus accumbens, and other brain regions. However, the neural oscillations sensed by the EEG were still very complex as it could not dissociate signals from deep brain areas and cortex after the emotion-related neural oscillations from the deep brain area were projected to the cerebral cortex. Therefore, it was necessary to encode and decode scalp EEG containing important emotional information efficiently. The spiking neural network algorithm could effectively simulate and restore the firing state of neurons uniquely in spike coding. This theory ensured the scientificity and reliability of the algorithm. Because the early SNN did not have an error compensation mechanism, it was plagued by accuracy for a long time. The SBP mechanism, first found in the hippocampal neurons, including long-term activation and long-term inhibition, strongly supported the backpropagation mechanism of the third-generation biological neural network. This mechanism significantly improved the calculation accuracy of the SNN. Because of the unique binary encoding mechanism, the calculation of model construction was greatly simplified, reducing the computer's energy consumption. Although we did not verify the advantages of computing cost in the research, previous studies have provided comprehensive and systematic proof (Zhang et al., 2021). Functional MRI and EEG technology are essential for studying brain activity and related mental diseases. However, compared with fMRI, the biggest shortcoming of EEG technology was low spatial resolution. The spatial resolution was mainly reflected in whether we could calibrate the spatial coordinate information of enough EEG nodes. The spatial coordinate of the whole-brain simulation is shown in Supplementary Figure S3. It could identify the precise spatial coordinate of the BSM of different emotions. Using spatial information in emotional research would play a more significant role in further study of the deep brain area of the emotional brain model using EEG.

There were significant differences in energy between emotional stimulus and neutral stimulus for both the time domain and time-frequency domain. This difference was concentrated in the late stage of clip playing, which might be related to the emotional cumulative effect of the video stimulation paradigm. This cumulative effect differed from the stimulation paradigm of pictures, sounds, slides, etc. From the perspective of brain processing on the stimulation, this process involved at least the neural pathway joint action of auditory and visual processing (Wang et al., 2014). It was still unclear what the transmission mode of the electrical activity in the brain was, especially how to find the existence of neural circuits through the EEG. The generation of emotion was with specific patterns of neural oscillatory. Previous studies proved a long pathway in the “emotional brain,” which was the critical link in generating emotion in the cerebral cortex. The electrical activity of the cerebral cortex of the emotions in the research was presented in a global brain network, which was widely distributed in the frontal, central, parietal, temporal, and occipital areas. Therefore, we could collect the neural spiking related to emotions in these brain areas. This result was consistent with the topological results of emotional brain activation (Supplementary Figure S2) and is in line with previous emotional studies based on fMRI (Liu et al., 2022). However, our comparative study of emotional subnetwork model performance showed that the prediction results of a single subnetwork model were superior to the global network, indicating a higher level of emotional brain network connection in the local subnetwork. Meanwhile, many low-level emotional brain network connections existed in the global network model, which led to an unsatisfactory result of the emotion prediction of the global fusion model. Meanwhile, the SFM based on single electrode sites lost too much emotional information and could not reflect the EEG effect of emotion well, which further proved the reliability of high-level connections in the local emotional brain network. Furthermore, how to analyze and make full use of low-level emotional information in the global emotional brain network needs further research.

The oscillation was a crucial biological marker to represent brain activity. The θ frequency band is essential to identify the emotion. However, our research found that the energy of the β band had better predictability for negative emotions, including fear and sadness; the α frequency band had a good prediction for happiness using the machine learning method. High-amplitude, regular α oscillation recorded from the occipital cortex represents relaxed wakefulness (resting condition). High cognitive load is represented by prolonged α oscillations at the frontal cortex (Oniz and Başar, 2009; Başar, 2012). β frequency band indicated a depressed and alert emotion state, which was widely used in psychological stress tests (Kumar and Kumar, 2016). Jenke et al. (2014) proved that α, β, and γ frequency bands might perform better using different EEG characteristics. This conclusion was consistent with our results: The middle- and high-frequency neural oscillation was an essential marker of emotional changes. The power of the β frequency band was proved to be related to negative emotions.

Therefore, α and β oscillations were selected as the biological marker of positive and negative emotions. However, because all the biological markers were β for negative emotions, we did not find a specific frequency band to distinguish specific negative emotions, such as fear and sadness. Therefore, it was necessary to do further research on the specific frequency band of a specific emotion. On this basis, the best local brain network model for different emotions was further established, which was a vital balance for the global fusion emotional brain network and the single electrode site fusion emotional brain network. This method not only compensated for the lack of emotion prediction accuracy of the single electrode sites fusion emotional brain network but also improved the sparsity of the global brain network, which was more conducive to the efficient evaluation of emotion. These results proved that a wide range of brain areas was involved. Furthermore, the local brain network could be used to decode emotion's neural activity more effectively comparing the global brain networks, which would provide an essential basis for the design of emotional regulation targets and signals.

Many methods were used to compute affection, statistics, machine learning, deep learning, and brain-like learning, such as support vector machine, k-nearest neighbor, logistic regression, decision tree, Naive Bayes, random forest, artificial neural network, convolutional neural networks, long short-term memory, and spiking neural networks. The power of α and β frequency bands as the emotional characteristic in the time-frequency domain was significantly better than that in the time domain in predicting emotion. α and β frequency bands were essential to identify real and fake happiness. The accuracies were 94.3% and 84.1%, respectively (Alex et al., 2020). We found that the accuracy of the α frequency band was better than that of the β frequency band in the prediction of happiness in the research. These results also supported our results on the α frequency band. The analysis of the DEAP dataset by Liu and Fu (2021) reached SROCC (Spearman rank-order correlation coefficient) of 0.789 and PLCC (Kendall rank-order correlation coefficient) of 0.843 with SVM being used for training the emotion. Deep learning models such as CNN, LSTM, and a hybrid of CNN-LSTM models were tested on the DEAP dataset. The investigation concluded that deep neural network has higher learning rate than other models and attained the best convergence with fewer epochs. This study obtained the best accuracy of 94.17% for the CNN-LSTM model (Zhang et al., 2020). In a model with a dynamic graph CNN for the classification of emotions from EEG signals using the SEED dataset was able to recognize with 90.4% accuracy for subject-dependent validation and 79.95% for subject-independent classification. Experiments conducted on the DREAMER dataset attained 86.23%, 84.54%, and 85.02% of average accuracies for valence, arousal, and dominance, respectively (Hasanzadeh et al., 2021). A study extracted entropy and Higuchi's fractal dimension features and applied empirical mode decomposition/intrinsic mode functions and variational mode decomposition on EEG signals. Their analysis of the DEAP dataset confirmed that CNN had better accuracy of 95.20% compared with naïve Bayes (92.27%), k-nearest neighbor (94.03%), and decision tree (88.50%) (Alhalaseh and Alasasfeh, 2020). The fusion algorithm of CNN and LSTM had an excellent performance. The prediction accuracies of emotion were 89.25%, 82.85%, and 89.38% for fear, sadness, and happiness. The prediction accuracy on the four-class classification was almost 70%. These results were much better than the algorithms of naive Bayes, k-nearest neighbor, decision tree, logistic regression, and SVM. The SNN represents the third generation of neural networks and employs biologically plausible models of neurons. SNN was used to decode the multimodal physiological signals and predict the valence of emotion. Its prediction accuracy had reached the same level as the deep learning algorithm (Tan et al., 2020). The SNN in the research was proved that it could be used to predict emotion, and the accuracy was above 80% for binary classifications. However, the performance was still lower than that of CNN-LSTM, mainly because the SNN algorithm only had forward propagation and no backward propagation mechanism, resulting in a model error that could not be eliminated. The prediction accuracy of different emotions was significantly improved after introducing the SBP mechanism (Zhang et al., 2021). In the prediction results of sadness, happiness, and four-class classifications, the performance of SBP-SNN exceeded the traditional machine learning and the CNN-LSTM, which also reminds us that combining the research of existing neural networks, the continuous introduction of new neurobiological mechanisms in spiking neural networks is conducive to improving the neural decoding performance of brain spired neural network algorithms, including accuracy, efficiency, and interpretability.

## Conclusion

To clarify the EEG brain network mechanisms of emotion, and to supply the intervention strategy of negative emotions, the spatiotemporal self-backpropagation spiking neural network with a biological mechanism was used to mine the EEG brain network of different emotions. The global and local emotional brain networks of fear, sadness, and happiness were established. The local brain network could better reflect the production of different emotions and significantly improve the sparsity of the emotional brain network. On this basis, it was found that the change of β and α band oscillations could better represent emotional changes, further clarifying the directional connection of each electrode node of the local emotional brain network. The critical elements of emotion recognition and intervention had been formed, including local emotional brain network nodes, connectivity, and biological markers. It would prepare for physical therapy for brain diseases. We increased the efficiency of the emotional brain network by improving the sparsity of the emotional brain network and biological encoding and decoding, which was consistent with the biological mechanism of low energy consumption and high accuracy of the brain network.

## Data availability statement

The raw data supporting the conclusions of this article will be made available by the authors, without undue reservation.

## Ethics statement

The studies involving human participants were reviewed and approved by Biomedical Ethics Committee, Peking University. The patients/participants provided their written informed consent to participate in this study.

## Author contributions

YX and WW conceived the study and designed the study. KC collected the data. HX and HC analyzed the data and wrote the manuscript. The contribution of HX, KC, and HC is equal. All authors contributed to the article and approved the submitted version.
