# Supplementary material for: Emotional brain network decoded by biological spiking neural network
Source: Front Neurosci. 2023 Jul 11;17:1200701. doi: 10.3389/fnins.2023.1200701 (PMC10366476; doi:10.3389/fnins.2023.1200701)
Supplement: Supplementary file 1 [file Data_Sheet_1.docx]

Supplementary Material

Article Title:Emotional brain network decoded by biological spiking neural network

Hubo Xu^1,2†^, Kexin Cao^1,2†^, Hongguang Chen^3†^, Awuti Abudusalamu^1,2^, Wei Wu^4*^, Yanxue Xue^1,5,6^^*^

*** Correspondence:** Corresponding Author: wei.wu@ia.ac.cn; yanxuexue@bjmu.edu.cn

# Supplementary Figures


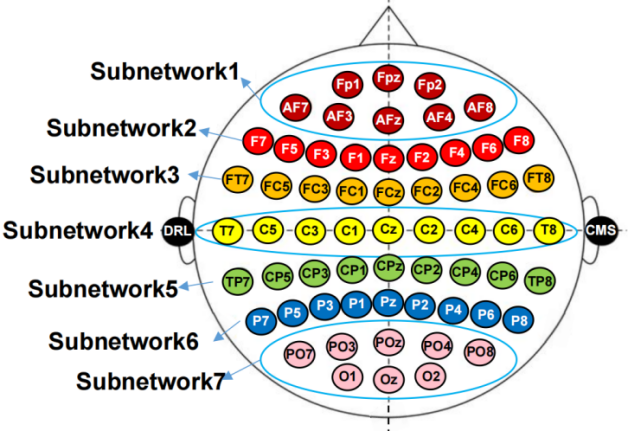


**FIGURE S1.** Spatial subnetwork division according to electrode distribution.


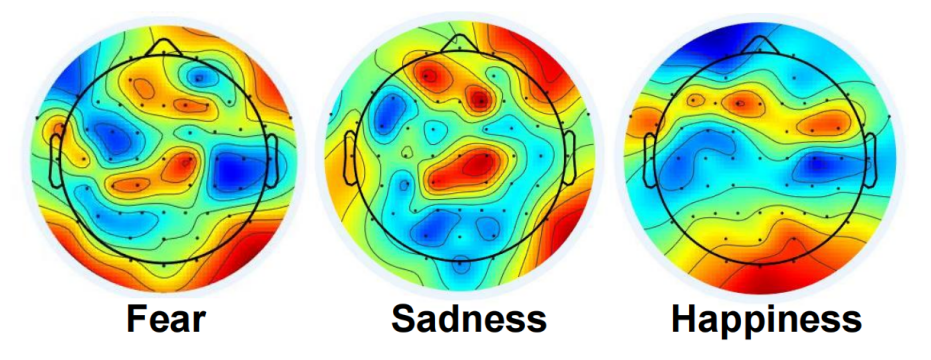


**FIGURE S2.** Topological energy distribution of emotional brain area.


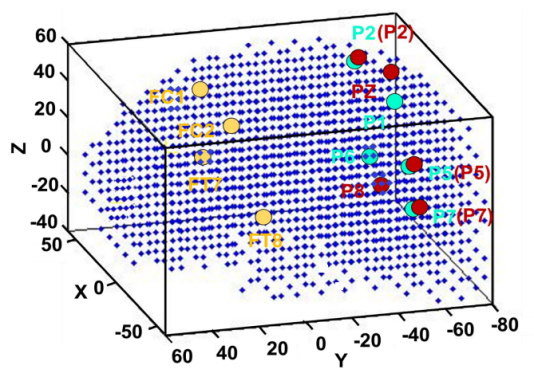


**FIGURE S2.** Spatial coordinates of the best subnetwork model, the yellow circle points represent the BSM of happy emotion, the blue circle points represent the BSM of fearful emotion, and the dark red circle points represent the BSM of sad emotion.

# Supplementary Tables

**TABLE S1.** Scores, affect intensity and hit rate of emotional video clips

| Video | Valence | Arousal | Dominance | Liking | Familiarity | Affect intensity | Hit rate |
| --- | --- | --- | --- | --- | --- | --- | --- |
| Neutrality 1 | 4.78±0.11 | 4.22±0.26 | 5.08±0.23 | 4.90±0.19 | 2.63±0.32 |  |  |
| Neutrality 2 | 4.63±0.13 | 3.88±0.27 | 4.90±0.21 | 4.61±0.16 | 2.02±0.25 |  |  |
| Happiness 1 | 7.33±0.18 | 6.71±0.18 | 5.90±0.24 | 7.08±0.21 | 7.08±0.31 | 6.65±0.29 | 97.96% |
| Happiness 2 | 7.45±0.19 | 6.80±0.23 | 5.90±0.23 | 7.00±0.19 | 3.41±0.39 | 6.73±0.33 | 95.92% |
| Sadness 1 | 3.29±0.18 | 5.69±0.21 | 4.71±0.20 | 4.65±0.21 | 2.71±0.34 | 5.06±0.31 | 93.88% |
| Sadness 2 | 2.98±0.21 | 6.00±0.20 | 4.76±0.25 | 4.59±0.24 | 2.37±0.30 | 5.59±0.34 | 93.88% |
| Fear 1 | 2.98±0.21 | 6.46±0.26 | 4.63±0.29 | 3.23±0.26 | 1.90±0.23 | 5.04±0.33 | 91.67% |
| Fear 2 | 2.59±0.23 | 7.47±0.20 | 4.04±0.33 | 3.16±0.32 | 1.88±0.21 | 6.41±0.32 | 93.88% |

**TABLE S2.** Performance of fear subnetwork models

| Subnetwork | Model 1 | | Model 2 | | Model 3 | | Model 4 | | Model 5 | | Model 6 | | Model 7 | |
| --- | --- | --- | --- | --- | --- | --- | --- | --- | --- | --- | --- | --- | --- | --- |
|  | Acc.  (%) | F1-Sc. | Acc.  (%) | F1-Sc. | Acc.  (%) | F1-Sc. | Acc.  (%) | F1-Sc. | Acc.  (%) | F1-Sc. | Acc.  (%) | F1-Sc. | Acc.  (%) | F1-Sc. |
| S1 | 59.00 | 0.59 | **75.00** | **0.77** | 72.73 | 0.73 | 65.91 | 0.62 | **72.73** | **0.74** | -- | -- | -- | -- |
| S2 | **56.82** | **0.57** | 50.00 | 0.47 | 54.55 | 0.49 | **50.00** | **0.49** | -- | -- | -- | -- | -- | -- |
| S3 | **54.55** | **0.55** | 50.00 | 0.49 | **47.73** | **0.47** | -- | -- | -- | -- | -- | -- | -- | -- |
| S4 | 65.91 | 0.63 | **77.27** | **0.74** | 59.09 | 0.54 | **65.91** | **0.73** | -- | -- | -- | -- | -- | -- |
| S5 | 61.36 | 0.65 | 65.91 | 0.63 | 63.64 | 0.63 | **72.73** | **0.71** | **68.18** | **0.68** | -- | -- | -- | -- |
| S6 | 77.27 | 0.79 | 79.55 | 0.82 | **81.82** | **0.84** | 75.00 | 0.76 | 81.82 | 0.83 | 70.45 | 0.74 | **72.73** | **0.74** |
| S7 | 75.00 | 0.74 | 77.27 | 0.78 | **79.55** | **0.81** | 65.91 | 0.68 | **63.64** | **0.65** | -- | -- | -- | -- |

**TABLE S3.** Performance of sadness subnetwork models

| Subnetwork | Model 1 | | Model 2 | | Model 3 | | Model 4 | | Model 5 | | Model 6 | | Model 7 | |
| --- | --- | --- | --- | --- | --- | --- | --- | --- | --- | --- | --- | --- | --- | --- |
|  | Acc.  (%) | F1-Sc. | Acc.  (%) | F1-Sc. | Acc.  (%) | F1-Sc. | Acc.  (%) | F1-Sc. | Acc.  (%) | F1-Sc. | Acc.  (%) | F1-Sc. | Acc.  (%) | F1-Sc. |
| S1 | 70.45 | 0.71 | 56.82 | 0.60 | 56.82 | 0.58 | **77.27** | **0.75** | **68.18** | **0.70** | -- | -- | -- | -- |
| S2 | 72.73 | 0.75 | **75.00** | **0.79** | 63.64 | 0.65 | 75.00 | 0.74 | **70.45** | **0.73** | -- | -- | -- | -- |
| S3 | 63.64 | 0.70 | 68.18 | 0.71 | **75.00** | **0.80** | 75.00 | 0.78 | 63.64 | 0.69 | **77.27** | **0.77** | -- | -- |
| S4 | 70.45 | 0.72 | 63.64 | 0.70 | 70.45 | 0.73 | **72.73** | **0.76** | 65.91 | 0.68 | 61.36 | 0.68 | **65.91** | **0.68** |
| S5 | 70.45 | 0.73 | **70.45** | **0.72** | 61.36 | 0.64 | 59.09 | 0.57 | **47.73** | **0.41** | -- | -- | -- | -- |
| S6 | 75.00 | 0.78 | 84.09 | 0.86 | **84.09** | **0.86** | 63.64 | 0.65 | 77.27 | 0.71 | 79.55 | 0.72 | **72.73** | **0.71** |
| S7 | 75.00 | 0.76 | 77.27 | 0.83 | 75.00 | 0.78 | **79.55** | **0.80** | **68.18** | **0.67** | -- | -- | -- | -- |

**TABLE S4** Performance of happiness subnetwork models

| Subnetwork | Model 1 | | Model 2 | | Model 3 | | Model 4 | | Model 5 | | Model 6 | | Model 7 | |
| --- | --- | --- | --- | --- | --- | --- | --- | --- | --- | --- | --- | --- | --- | --- |
|  | Acc.  (%) | F1-Sc. | Acc.  (%) | F1-Sc. | Acc.  (%) | F1-Sc. | Acc.  (%) | F1-Sc. | Acc.  (%) | F1-Sc. | Acc.  (%) | F1-Sc. | Acc.  (%) | F1-Sc. |
| S1 | 45.45 | 0.45 | 50.00 | 0.42 | **56.82** | **0.54** | **40.91** | **0.43** | -- | -- | -- | -- | -- | -- |
| S2 | 61.36 | 0.62 | 72.73 | 0.75 | **77.27** | **0.79** | 61.36 | 0.65 | 72.73 | 0.71 | 72.73 | 0.71 | **68.18** | **0.70** |
| S3 | 75.00 | 0.78 | 68.18 | 0.70 | **81.82** | **0.83** | 77.27 | 0.81 | 75.00 | 0.78 | **79.55** | **0.80** | -- | -- |
| S4 | **81.82** | **0.83** | 79.55 | 0.79 | 59.09 | 0.69 | 68.18 | 0.72 | 68.18 | 0.72 | 72.73 | 0.76 | **63.64** | **0.56** |
| S5 | **75.00** | **0.77** | 54.55 | 0.50 | 63.64 | 0.64 | 65.91 | 0.65 | **56.82** | **0.56** | -- | -- | -- | -- |
| S6 | 79.55 | 0.82 | **79.55** | **0.82** | 72.73 | 0.75 | 72.73 | 0.73 | 68.18 | 0.72 | 70.45 | 0.72 | **65.91** | **0.68** |
| S7 | 72.73 | 0.71 | 77.27 | 0.74 | **79.55** | **0.81** | 72.73 | 0.74 | **72.73** | **0.71** | -- | -- | -- | -- |
